# Evaluation of cost of treatment of malaria in adults in Benin City, Nigeria: patients’ perspective

**DOI:** 10.5281/zenodo.10818212

**Published:** 2016-09-16

**Authors:** Obumneke A. Obieche, Valentine U. Odili

**Affiliations:** 1 Department of Clinical Pharmacy and Pharmacy Practice, University of Benin, Benin City, Edo State, Nigeria

## Abstract

**Background:**

Malaria remains a disease of immense clinical and economic significance. Limited research has been carried out to estimate malaria treatment costs at the health care facility level using the patient’s perspective. The objectives of this study were therefore to determine the direct and indirect costs of malaria treatment among adult outpatients and to assess the patients’ perception of treatment costs.

**Materials and methods:**

A cr oss-sectional study was conducted at the Pharmacy section of the General Practice Clinic, University of Benin Teaching Hospital, Benin City, Edo State, Nigeria. It involved adult outpatients diagnosed with malaria and who received a prescription of one or more anti-malarial medications. A cost-of-illness approach was employed in the assessment of costs of treatment of malaria per sick adult patient. Pre-tested semi-structured questionnaires were used in the study. Furthermore, self-reported incidence of malaria per year was assessed.

**Results:**

The mean direct and indirect cost of tr eating malaria illness per adult outpatient was Nigerian Naira (NGN) 3417.70 ($ 20.34) and NGN 4870 ($ 29.0), respectively, giving a ratio of 0.7:1. Medications and laboratory tests for detection of malaria parasites contributed about 52 and 22% of the total direct cost, respectively. A total of 1592 malaria episodes were self-reported to occur annually, giving a mean value of 3.35 episodes per adult. Having a health care insurance was associated with the response that the cost of malaria treatment was low (*P*< 0.001).

**Conclusion:**

The mean values of direct cost and indirect cost of treatment of malaria illness per adult outpatient were $ 20.34 and $ 29.0, respectively. Respondents who had health insurance perceived malaria treatment cost to be low, whereas those without such insurance felt otherwise.

## 1 Introduction

Malaria is an important cause of morbidity and mortality in individuals living in tropical countries. It is a major public health concern in Nigeria, and poses a leading cause of illness, hospitalisation and death in Nigeria [1]. This is consistent with the fact that malaria is holo-endemic in Nigeria, with 97% of Nigeria’s population at risk of contracting the disease [2,3]. Globally, it is estimated that 214 million clinical cases of malaria (range 149-303 million) occurred in 2015 [4]. Available records show that there are an estimated 100 million malaria cases per year in Nigeria [2]. Every year, ~50% of Nigerian adults suffer at least one episode of malaria [5], while children under 5 years of age have an average of 2-4 bouts of malaria [6].

Huge resources are spent on malaria case management, selective vector control, intermittent prophylaxis during pregnancy, and epidemic detection and control. The amount spent on malaria in terms of prevention, treatment and loss of productivity can comprise a significant proportion of the annual income of poor households [7]. In a study conducted in rural communities in Nigeria, Onwuje-kwe *et al.* [8] reported that the cost of treating malaria illness by households accounted for 49.9 % of curative health care costs incurred by the households, which amounted to an average malaria expenditure of $ 1.84 per household per month. Yet, in another Nigerian study conducted in 2007, which used a willingness-to-pay approach, households were found to be willing to pay an average of about NGN 1,112 ($ 9.3) per month for the treatment of malaria rather than bear the burden of the disease on their physical, mental, and social well-being [9]. This was further supported by results of a later study, which showed that households in Africa spend $ 2-25 on malaria treatment and $ 15-20 on preventive measures monthly, with consequent loss of resources [10].

The economic burden of malaria illness can be assessed using cost-of-illness or willingness-to-pay methods. Both methods have been explored in a variety of studies [9,11-14]. The cost of treatment of malaria may be evaluated using a cost-of-illness approach. With this method of evaluation, the direct costs of an illness or a disease condition are the costs of treatment as well as control interventions. These include cost of hospital registration cards, medication cost, cost of investigation, and other costs incurred as a result of seeking treatment for the disease. The costs are further subdivided into direct medical cost and direct non-medical costs. The indirect costs are determined as productivity losses due to malaria illness, as measured by income foregone due to morbidity, disability and mortality [13]. Simply put, indirect cost involves evaluating decline in productivity and reduction of income through lost time during the period of illness. Absence from work can lead to significant income losses, particularly in privately owned businesses where income is largely dependent on the number of productive hours.

Significant financial losses have been reported across African economies, such as Uganda and Ethiopia, of which a large proportion was incurred by households or individuals through out-of-pocket payment and or productivity loss due to person-days lost [15,16]. Numerous studies have been carried out to estimate treatment costs of malaria at household levels but only a few of these studies prospectively assessed the costs of treatment from a patients’ perspective [17]. A prospective study would mini-mise errors due to recall bias that might be evidenced while feeding in information on the costs of malaria illness. The objectives of this study were thus to determine the direct and indirect costs of treatment of malaria among adult outpatients and to assess patients’ perception regarding the treatment costs.

## 2 Materials and methods

### 2.1 Study design, location and population

A prospective, cross-sectional study was designed to assess the direct and indirect costs of treatment of uncomplicated malaria from the patient’s perspective at the pharmacy section of the General Practice Clinic, University of Benin Teaching Hospital, Benin City, Edo State, Nigeria. The study was carried out between September and November 2014. Participants were adult outpatients (18 years and older) who had a positive malaria diagnosis either clinically or by parasite-based methods, and were given prescription orders for anti-malarial medication and other medication related to malaria-associated symptoms. All eligible participants were identified while they were waiting for their medications and counselling with the pharmacists. Subsequently, they were approached and given information regarding the study objectives and were assured of confidentiality of personal information. Excluded from the study were children under the age of 18 years, patients with chronic co-morbidities and those who did not give consent for the study. Any adult patient who had been interviewed earlier in the study was excluded on subsequent hospital visits.

### 2.2 Sample size

With a 17% prevalence of malaria in Nigerian adults [18], the sample size was determined as 469. In view of possible loss of data due to, for example, inappropriately filled out questionnaires, an additional 4% of the determined sample size was included.

### 2.3 Data collection

Semi-structured questionnaires were used. Prior to use these were pretested at the study site using 50 adult patients diagnosed malaria-positive either clinically or by parasite-based methods and who came for filling of their anti-malarial medications. The questionnaires consisted of four sections. The first section of the questionnaire sought the socio-demographic characteristics of the study participants, such as gender, age, occupation, income, education and health insurance. The second, third and fourth sections assessed the indirect costs of treatment of malaria, direct costs of treatment of malaria, and patients’ perceptions about the cost of treatment of malaria illness, respectively. Also, under patient’s perception, self-reported incidence of malaria per year was assessed. In addition, the indirect cost section had a scale ranging from 0 to 100% (5%-intervals) drawn by the side of the questionnaire. All respondents who reported that they would go back to their places of work after collecting medication at the pharmacy section were asked to rate their possible efficiency when they eventually got back to work. The questionnaires were self-administered to those who were literate, whereas non-literate respondents were interviewed by the researcher. The information given by the patients with regard to costs of medication was complemented with data abstracted from the prescription orders.

### 2.4 Data analysis

Direct medical costs were computed by evaluating the costs of hospital registration cards, medications, and laboratory investigations related to malaria illness. Where available, receipts of purchases were crosschecked with verbally reported figures. Similarly, direct non-medical cost evaluated the transport fare/travel cost incurred by the patient and caregiver, if any. Assessment of indirect costs estimated the productivity losses due to reduction of income during the worker’s (patient’s) incapacitation. In estimating the indirect cost, the monetary values of absenteeism at work were determined. Firstly, the average daily income (ADI) of the patient was determined. Next, the number of days that patients were absent from work due to malaria illness (DA) was obtained. This was determined as the number of days prior to the day of interview that patients have been absent from work due to the current malaria illness plus one day (representing the day of interview at the clinic) plus ‘sick-off’ days given to the patient by the physician. The product of ADI and DA was calculated to give an estimate of productivity loss [18].

Data was entered into Microsoft Excel data sheets, crosschecked, uploaded and analysed using SPSS for Windows version 16.0. Descriptive statistics were used for continuous variables (means ± SD), whereas categorical variables were presented as frequencies and percentages. Chi-square test (*X ^2^*) was used to investigate whether distributions of categorical variables differ from one another, while between-group comparisons were evaluated by Student’s unpaired t-test. *P*-values <0.05 were considered significant.

### 2.5 Ethical considerations

Written and or verbal informed consent was obtained from each study participant before the questionnaires were administered to them.

## 3 Results

A total of 528 patients had prescription orders meant for treatment of malaria and malaria-related symptoms. Of these, 487 agreed to participate in the study and were each administered the questionnaire. Four-hundred and seventy-five (97.5%) of the retrieved questionnaires were found valid and were therefore used in the analysis.

### 3.1 Sociodemographic data of the study participants

The age of the respondents ranged from 18 to 75 years, with a mean age of 38.8±4.6 years. The majority of the respondents were female (311, 65.5%), married with live spouses (339, 70%) and had tertiary level of education (346, 72.8%) (Table 1). The majority (344, 72.4%) of the study participants were registered with a health insurance scheme and most of them (336, 70.7%) were government-employed workers. The mean income per month was NGN 79,254.60±60,724, determined for only 398 respondents that indicated the value of their monthly income. Only 69 (14.5%) patients reported to have come with a caregiver. Further socio-demographic details are shown in Table 1.

**Table 1.** Socio-demographic characteristics of respondents.

Variable	Number	%
**Age (years)**		
18 – 25	56	11.8
26 – 30	59	12.4
31-35	100	21.1
36-40	88	18.5
> 40	172	36.2
**Gender**		
Male	164	34.5
Female	311	65.5
**Level of education**		
Primary	45	9.5
Secondary	84	17.7
Tertiary	346	72.8
**Marital status**		
Single	108	22.7
Married with live spouse	339	71.4
Living with a partner	18	3.8
Divorced/widowed	10	2.1
**Health insurance scheme**		
Yes	344	72.4
No	131	27.6
**Primary source of income**		
Employed by government	336	70.7
Self-employed	54	11.4
Privately employed	16	3.4
Unemployed	38	8
Family relatives	31	6.5
**Income/allowance per month (NGN)**		
< 10, 000	28	5.9
> 10, 000 – 30, 000	35	7.4
> 30, 000 – 50, 000	108	22.7
> 50, 000 – 70, 000	73	15.4
> 70, 000 – 90, 000	21	4.4
> 90, 000 – 100, 000	28	5.9
> 100, 000 – 150, 000	41	8.6
> 150, 000 – 200, 000	36	7.6
> 200, 000 – 250, 000	22	4.6
> 250, 000 – 300, 000	6	1.3
Missing	77	16.2
**Patient that came with caregiver**		
Caregiver	69	14.5
No caregiver	406	85.5

### 3.2 Patient-reported malaria cases and their perceptions about the cost of malaria treatment

All study participants reported that they had at least one episode of malaria illness per year; this ranged from 1 to 8 episodes per patient per year (Table 2). More than 85% (412) of the respondents said that they usually had more than one case of malaria in a year. A total of 1582 malaria episodes were self-reported to occur annually, giving a mean value of 3.33 malaria cases per adult patient. On the issue of whether patients sought immediate professional medical attention for perceived malaria illness, 78% (371) gave an affirmative response, while the remaining proportion of 22% (104) said that they often practiced self-medication and then sought appropriate medical care when symptoms persisted.

**Table 2.** Frequency distribution of occurrence of malaria cases per year among adult patients.

Episodes/ year	Number of patients	%	Subtotal of cases
1	63	13.3	63
2	88	18.5	176
3	110	23.2	330
4	116	24.4	464
5	60	12.6	300
6	21	4.4	126
7	13	2.7	91
8	4	0.8	32
Total	475	99.9	1582

Slightly more than 50% (254) of the study participants reported that the cost of malaria treatment was low and that it did not impact greatly on their financial resources, while the remaining 46.5% (221) respondents indicated that the treatment cost was high. The mean values of income per month for those patients that reported low and high cost of malaria treatment were NGN 103,107.20 and NGN 54,545.0, respectively (*P*<0.001). Of all the participants on health insurance, 62.8% (216/344) said that treatment cost of malaria illness did not affect their financial savings compared to 37.2% (128/344) who felt contrary (*X ^2^*= 43.521, *P*<0.001; Table 3).

**Table 3.** Comparison of patient opinion on the cost of treatment of malaria in respect to their status of health insurance (*X ^2^* = 43.521, *P* < 0.001).

	Patient opinion treatment of on cost of malaria		
Health care insurance?	High	Low	Total
**Yes**	128	216	344
**No**	93	38	131
**Total**	221	254	475

### 3.3 Direct costs of malaria treatment

The mean cost of medication was NGN 1760.50±933.54 ($ 10.48±5.56). The costs of hospital registration card plus administrative services and laboratory-based diagnostic methods for malaria (microscopic examination of stained blood films) were NGN 600.00 ($ 3.57) and NGN 750.00 ($ 4.46), respectively. Malaria parasite tests were carried out in 166 (35%) of patients. The mean value of the direct medical costs among the study participants was NGN 3110.50 ($ 18.51). The cost of transportation/travel among patients who used commercial means of transportation (that is, direct non-medical cost) was NGN 307.20±244.46 ($ 1.83±1.46). Overall, the mean total direct cost of malaria treatment was NGN 3417.70 ($ 20.34).

The cost of medications used in the treatment of malaria and malaria-related symptoms constituted about 51.5 % of the total direct cost of malaria treatment. On the other hand, costs of hospital registration card plus administrative services and laboratory investigation for malaria parasites contributed 17.6 and 22%, respectively, totalling about 40% of the total direct cost of malaria treatment.

### 3.4 Prescribed medications

Table 4 shows the various medications that were prescribed and dispensed to the respondents for the treatment of malaria. The most frequently prescribed antimalarials were artemisinin-lumefantrine (89.1%) and artemisinin-mefloquine (6.5%). Paracetamol (69.1%), multivitamins (21.3%), vitamin C (14.5%), chlorpheniramine (17.3%) and diclofenac (11.4%) were the most frequently prescribed non-antimalarial medications intended for treatment of malaria-related symptoms. Brand prescription and brand dispensing were recorded for the antimalarial medications, except for quinine.

**Table 4.** Various antimalarial and non-antimalarial medications prescribed for the treatment of malaria illness.

Medication prescribed	Number of patients	%
**Antimalarial medications**		
Artemisinin-lumefantrine	423	89.1
Artemisinin-mefloquine	31	6.5
Quinine	12	2.5
Sulphadoxine-pyrimethamine	9	1.9
**Non-antimalarial medications**		
Paracetamol	328	69.1
Multivitamins	101	21.3
Vitamin C	69	14.5
Promethazine	37	7.8
Cough syrup	51	10.7
Chlorpheniramine	82	17.3
Loratidine	48	10.1
Diclofenac Na	54	11.4
Nitrazepam	18	3.8
Diazepam	10	2.1

### 3.5 Indirect costs of malaria treatment

An assessment of indirect cost of treatment of malaria showed that more than 50% (254) of the patients spent 2-4 hours at the hospital before they got to the pharmacy for their medications. Table 5 shows the number of days lost (absence from work) due to malaria illness and the number of hours spent while waiting for medical consultation/ laboratory test results. Absence from work was recorded in 198 patients (42%), and the number of days of absenteeism ranged from 1 to 6 days. Overall, a total of 398 days is estimated to be lost during the participants’ current malaria illness. Of the 167 respondents who stated their income, the sum of NGN 813,290 was calculated as the loss in productivity during their days of incapacitation (absence from work). This constituted the indirect cost of treatment of malaria, giving a mean value of NGN 4,870 ($ 29.0) per respondent.

**Table 5.** Number of days lost due to malaria illness and waiting hours encountered in seeking health care services for malaria treatment.

Indirect non-medical cost	Number of patients	%
**Absenteeism (days)**		
0	277	58.3
1	93	19.6
2	55	11.6
3	24	5.1
4	11	2.3
5	11	2.3
6	4	0.8
**Waiting (hours)**		
< 1	23	4.8
1 – 2	66	13.9
2 – 3	87	18.3
3 – 4	167	35.2
> 4	132	27.8

More than half the participants (58%) said that they would go back to their work places after receiving medication at the outpatient pharmacy. When asked to rate their possible efficiency on return to their work schedule, none of them rated their efficiency above 70%, while 68 (24.5%), 124 (44.8%) and 85 (30.7 %) respondents reported varying ranges of efficiency: < 50%, 50-60%, and 6570%, respectively.

## 4 Discussion

This study showed that the indirect cost of malaria treatment was higher than the direct cost. Slightly more than half of the study participants agreed that the cost of malaria treatment does not impact significantly on their financial resources, yet having a health insurance further supports this perception.

The incidence of malarial illness is notably high even for Nigerian adults who are believed to have built up sufficient immunity. In a similar study involving the interview of 1200 Nigerian households, 81 and 18% of the households recorded 1 and 2 malaria episodes respectively within the reference period of one month while a range of 1-6 malaria cases per household per month was reported [11].

There was divided opinion among the adult patients on whether the treatment cost of malaria was high or low. Also, significant difference existed between the mean income per month for those patients that reported low and high costs of malaria treatment. Patients that perceived that the cost of treating malaria illness was high had lower mean income value. This supports results of earlier studies that showed that costs of malaria illness varied with socioeconomic status, where the poor tended to spend a considerable portion of their incomes on the treatment of malaria [19,20]. In addition, having or not having a health care insurance influenced perception to cost of malaria treatment. At present, the National Health Insurance Scheme (NHIS) is not yet fully implemented in the country. Only 10% of total cost of medications (listed in the health insurance scheme) is paid by the insured patients with no deductions yet in their incomes to offset the remaining huge proportion (90%) of the medication cost. Thus, the patients are benefitting from a highly subsidised medical care and as such would perceive cost of medical care to be low. It is therefore possible that these patients might change their views about treatment cost of malaria when they begin paying appropriate premiums due to their health insurance.

In the present study, the indirect costs (i.e. productivity losses) of treatment of malaria contributed up to 59% of total malaria treatment costs. Even higher percentages of 72 and 79% have been recorded for indirect costs in other previous studies done in Ethiopia and Ghana, respectively [16,21]. A community-based cross-sectional study done in rural Southcentral Ethiopia that employed cost of illness approach reported an average estimated direct cost of malaria treatment per patient of $ 1.60 while the average indirect cost was $ 4.08 [16]. The high indirect costs recorded in our study, in part, resulted from considerable ‘sick-off’ days given to the patients by the medical practitioners. Again, some of the patients involved in this study spent few days practicing self-medication and being away from work, which were counted for them when they eventually enrolled for the study. In another study, an average of 3 days was lost by a sick adult due to malaria illness [11]. Moreover, the high indirect costs may be explained by the fact that concerted efforts have been made over the years toward reducing direct costs of treatment of malaria by provision of free and highly subsidised anti-malarial drugs. Indeed, patients are still left with enormous costs of laboratory tests and transportation as well as productivity loss. These patient-borne costs have been highlighted in a previous study where patients spend significant amounts of money on transportation and food/snacks consumed while waiting for medical attention, and also lose resources through productivity loss [22]. Presence at workplaces as practiced by a majority of the study participants may not necessarily translate to optimal output in services, as there would be decreased efficiency. It might be beneficial to the workers as well as the organisation if a sick worker is duly granted absence from duty for a reasonable number of days in order to ensure proper treatment and quick recovery.

Even though the mean income obtained in this study was NGN 79,254.60 (± NGN 60,724), the treatment costs of malaria illness could consume a significant portion of the patients’ income, particularly those whose incomes lie at the lower level of the income range.

In the present study, there was evidence of presumptive treatment of malaria. This might have led to over-diagnosis of the disease, which was reported in previous studies [23,24]. The implication of this trend is gross wastage of scarce resources on anti-malarial medications. Moreover, there would be a delay in initiating the correct treatment in those patients who did not have malaria. It was also noted that patients spent a lot of time in the hospital while seeking treatment for malaria, and this was even higher among those that had laboratory tests for malaria. The use of rapid diagnostic tests (RDTs) would reduce the number of hours such patients spend in the hospital, as the tests and results could be completed within a few minutes. Extensive prescription of artemisinin-based combination therapy, typically artemisinin-lumefantrine, was recorded at the study site. This is in line with the WHO treatment guidelines for uncomplicated malaria [25]. However, brand prescribing of antimalarial medications was evident in most of the prescriptions. Undoubtedly, this practice increased the costs of medication used in malaria treatment. Low occurrence of generic prescribing of antimalarial drugs had earlier been reported from Nigeria [26-28]. Generic medications are generally cheaper than brand products. Thus, generic prescribing and generic substitution have been advocated as possible mechanisms for cost reduction in health care. However, concerns about the quality and efficacy of these generic substitutes will have to be managed.

In conclusion, the treatment of malaria in adults is associated with significant costs, particularly for the low-income earners. The mean direct cost and indirect cost of treatment of malaria for an adult outpatient were NGN 3417.70 ($20.34) and NGN 4870 ($28.98), respectively, giving a total cost of managing a malaria episode of NGN 8287.70 ($49.34). This is much higher than the median financial cost of $5.84 (range $2.36-$23.65) for treatment of uncomplicated malaria reported in a systematic review of the costs and cost effectiveness of malaria control interventions [29]. Medications accounted for 51.5% of the total direct costs, and this high value is attributed to extensive brand prescribing practiced at the study site. More than 50% of the respondents agreed that the cost of malaria treatment did not impact significantly on their financial resources. This was particularly so for those who had a health insurance cover. The findings of this study show that, despite the scale-up of malaria interventions, which include donation of artemisinin-based combination therapies (ACTs) for all ages [30], malaria still places a heavy financial burden on low-income earners of countries like Nigeria, where the national minimum wage is NGN 18,000.00 ($107.14).

A limitation of the present study was the recall bias that might be present during the interview, as some patients might not have accurately reported the annual frequency of malaria. Again, in calculating the indirect cost (productivity loss), the number of ‘sick off’ days given by the medical practitioners were used. There is a possibility that some patients, particularly those who engage in private businesses, may go back to their businesses without exhausting the ‘sick off’ days. The costs of possible further treatment such as may occur with treatment failure with regard to the present illness was not accounted for.

## 5 Conclusion

The mean values of direct cost and indirect cost of treatment of malaria illness per adult outpatient were $ 20.34 and $ 29.0, respectively. Respondents who had health insurance perceived malaria treatment cost to be low, whereas those without such insurance felt otherwise.

## References

[2] http://nigeria.usembassy.gov.

[6] Adedotun AA, Morenikeji OA, Odaibo AB (2010). Knowledge, attitudes and practices about malaria in an urban community in south-western Nigeria.. J. Vector Borne Dis..

[7] Chima R, Goodman CA, Mills A (2003). The economic impact of malaria in Africa: a critical review of the evidence.. Health Policy.

[8] Onwujekwe OE, Chima R, Okonkwo PO (2000). The economic burden of malaria illness versus that of a combination of all other illnesses: A study in five malaria holo-endemic communities.. Health Policy.

[9] Jimoh A, Sofola O, Petu A, Okorosobo T (2007). Quantifying the economic burden of malaria in Nigeria, using willingness to pay approach.. Cost. Eff. Resour. Alloc..

[10] Oluyole KA, Ogunlade MO, Agbeniyi SO (2011). Socioeconomic burden of malaria disease on farm income among cocoa farming households in Nigeria.. American-Eurasian J. Agric. Environ. Science.

[11] Salihu OM, Sanni NA (2013). Malaria burden and the effectiveness of malaria control measures in Nigeria: A case study of Asa Local government area of Kwara State.. J. Econ. Sust. Dev..

[12] Onwujekwe O, Hanson K, Fox-Rushby J (2004). Inequalities in purchases of mosquito nets and willingness to pay for insecticides treated net in Nigeria.. Malar. J..

[13] Alaba O, Alaba A (2006). Malaria in rural Nigeria: Implication for the Millennium Development Goal.. African Economic Research Consortium..

[14] Shepard DS, Ettling MB, Brinkmann U, Sauerborn R (1991). The economic cost of malaria in Africa.. Trop. Med. Parasitol..

[15] Nabyonga OJ, Muthuri KJ, Azairwe R, Muheki ZC (2011). Cost of malaria morbidity in Uganda.. J. Biol. Agric. Healthc..

[16] Deressa W, Hailemariam D, Ali A (2007). Economic costs of epidemic malaria to households in rural Ethiopia.. Trop. Med. Int. Health..

[17] Onwujekwe O, Uguru N, Etiaba E, Chikezie I (2013). The economic burden of malaria on households and the health system in Enugu State southeast Nigeria.. PLoS One.

[18] Anumudu CI, Adepoju A, Adeniran M, Adeoye O (2006). Malaria prevalence and treatment seeking behaviour of young Nigerian adults.. Ann. Afr. Med..

[19] Sharma SK, Pradhan P, Padhi DM (2001). Socioeconomic factors associated with malaria in a tribal area of Orissa, India.. Indian J. Public Health..

[20] Sauerborn R, Shepard DS, Ettling MB, Brinkmann U (1991). Estimating the direct and indirect cost of malaria in a rural district of Burkina Faso.. Trop. Med. Parasitol..

[21] Asenso-Okyere WK, Dzatorb JA (1997). Household cost of seeking malaria care. A retrospective study of two districts in Ghana.. Soc. Sci. Med..

[22] Bruno M, Damme W, Tashobya C, Tibouti A (2006). Poverty and user fees for public health care in low-income countries: lessons from Uganda and Cambodia.. Lancet.

[23] Ndyomugyenyi R, Magnussen P, Clarke S (2007). Diagnosis and treatment of malaria in peripheral health facilities in Uganda: findings from an area of low transmission in South-Western Uganda.. Malar. J..

[24] Oladosu OO, Oyibo WA (2013). Over diagnosis and overtreat-ment of malaria in children that presented with fever in Lagos, Nigeria.. ISRN Infectious Diseases..

[25] World Health Organization: World Health Organization guidelines for the treatment of malaria. (2006). World Health Organization, Geneva..

[26] Akande TM, Ologe MO (2007). Prescription pattern at a secondary health care facility in Ilorin, Nigeria.. Ann Afr. Med..

[27] Isah AO, Laing R, Quick J, Mabadejele A.F.B, Santos B, Hogerzeil H, Ross-Degnan D (2002). The Development of Reference Values for the WHO Health Facility Core Prescribing Indicators West Afr.. J. Pharmacol. Drug Res..

[28] Igboeli NU, Ukwe CV, Ekwunife OI (2010). Increasing use of artemisinin-based combination therapy for treatment of malaria infection in Nigerian hospitals.. Pharmacy Pract..

[29] White MT, Conteh L, Cibulskis R, Ghani AC (2011). Costs and cost-effectiveness of malaria control interventions - a systematic review.. Malar J..

[30] http://www.who.int/malaria/media/world-malaria-report-2015/en/.

